# Physicochemical characterization of ferric pyrophosphate citrate

**DOI:** 10.1007/s10534-018-0151-1

**Published:** 2018-10-15

**Authors:** Ajay Gupta, Raymond Pratt, Bhoopesh Mishra

**Affiliations:** 1 University of California Irvine, Orange, CA USA; 2 Rockwell Medical, Wixom, MI USA; 3 University of Illinois College of Medicine, Chicago, IL USA; 4 Physics Department, Illinois Institute of Technology, Chicago, IL USA; 5 School of Chemical and Process Engineering, University of Leeds, Leeds, West Yorkshire LS2 9JT UK

**Keywords:** Ferric pyrophosphate citrate, XAFS, Synthesis, Physicochemistry

## Abstract

Iron deficiency is a significant health problem across the world. While many patients benefit from oral iron supplements, some, including those on hemodialysis require intravenous iron therapy to maintain adequate iron levels. Until recently, all iron compounds suitable for parenteral administration were colloidal iron–carbohydrate conjugates that require uptake and processing by macrophages. These compounds are associated with variable risk of anaphylaxis, oxidative stress, and inflammation, depending on their physicochemical characteristics. Ferric pyrophosphate citrate (FPC) is a novel iron compound that was approved for parenteral administration by US Food and Drug Administration in 2015. Here we report the physicochemical characteristics of FPC. FPC is a noncolloidal, highly water soluble, complex iron salt that does not contain a carbohydrate moiety. X-ray absorption spectroscopy data indicate that FPC consists of iron (III) complexed with one pyrophosphate and two citrate molecules in the solid state. This structure is preserved in solution and stable for several months, rendering it suitable for pharmaceutical applications in solid or solution state.

**Electronic supplementary material:**

The online version of this article (10.1007/s10534-018-0151-1) contains supplementary material, which is available to authorized users.

## Introduction

Iron deficiency with or without associated anemia represents a significant health problem worldwide. While many patients can restore iron levels with the use of oral iron supplements, oral supplementation is not suitable in some patients, including those undergoing chronic hemodialysis for chronic kidney disease (CKD) (Fudin et al. 1998; Macdougall et al. 1996; Markowitz et al. 1997). The limitations of oral iron replacement in patients undergoing hemodialysis likely arise from excessive ongoing losses and insufficient absorption, thus intravenous (IV) iron has become the primary route of administration in such patients (Shah et al. 2016). Multiple IV iron formulations are available, including iron dextran, iron sucrose, sodium ferric gluconate, iron carboxymaltose, ferrumoxytol, and iron isomaltoside (Macdougall et al. 1996). All such formulations are iron–carbohydrate macromolecular complexes, and the majority consist of an iron oxide core surrounded by a carbohydrate moiety (Macdougall et al. 1996; Markowitz et al. 1997).

Intravenous iron products have been used extensively for over 30 years for the treatment of iron-deficiency anemia and to maintain iron balance in hemodialysis patients since these patients have obligatory excessive losses. While these agents are generally well tolerated, they have been associated with risk of anaphylaxis (Wang et al. 2015). Compared to oral iron agents, there may be an increased risk of cardiovascular complications and infections in nondialysis patients with CKD (Macdougall et al. 1996). Additionally, higher mortality rates have been reported with use of high-dose IV iron in hemodialysis patients (Bailie et al. 2015).

Iron possesses oxidizing properties that may cause injury to cells and tissues (Koskenkorva-Frank et al. 2013; Vaziri 2013). Iron loading in general is associated with endocrinological, gastrointestinal, infectious, neoplastic, neurodegenerative, obstetric, ophthalmic, orthopedic, pulmonary, and vascular complications. In addition, excessive or misplaced tissue iron also can contribute to aging and mortality (Weinberg 2010). Normally, the body is able to protect tissues from the damaging effects of iron by regulating iron absorption in the intestine and sequestering iron with iron-binding proteins. However, the concentrations of iron introduced into the bloodstream with IV iron therapy can be as much as 100 times more than that absorbed normally through the intestine. Combined with the fact that IV iron is administered over a period of minutes compared to the slow, regulated absorption in the gut, it is possible that the increased iron load may damage cells and tissues.

A novel parenteral iron formulation, ferric pyrophosphate citrate (FPC), potentially offers a more physiologic delivery of iron. Unlike previous forms of IV iron, FPC contains no carbohydrate shell. Soluble ferric pyrophosphate-citrate complexes, generally referred to as soluble ferric pyrophosphate (SFP) were first described in the mid-1800s by Robiquet and Chapman (Chapman 1862; Robiquet 1857). This class of food-grade iron salts has been available for over 100 years as oral iron supplements and for fortification of food. In the late-1990s, Gupta et al. demonstrated that food-grade SFP could be administered to hemodialysis patients via the dialysate (Gupta et al. 1999). However, the commercially available compounds are poorly characterized and not suitable for further development as a parenteral iron supplement. Therefore, a pharmaceutical-grade SFP was developed. This product had a higher solubility than food-grade SFP and was granted a new USAN name—FPC. In 2015 FPC was approved by the US Food and Drug Administration (FDA) for parenteral delivery by hemodialysis to replace iron losses and thereby maintain hemoglobin levels in hemodialysis-dependent patients with CKD (Rockwell Medical Inc 2018). FPC is currently marketed under the trade name Triferic^®^ (Rockwell Medical Inc., Wixom, Michigan, USA). FPC is the first carbohydrate-free, noncolloidal, water-soluble iron salt suitable for parenteral administration.

This study aimed to determine the solid- and solution-phase characteristics of FPC, including the coordination environment of iron and its neighboring atoms, and to evaluate the stability of FPC in solution. The results presented here describe the physicochemical characterization of FPC in the solid state and in solution and characterize the unique iron (III)-citrate-pyrophosphate ternary complex oligomeric structure, which is stable for extended periods in aqueous solution.

## Materials and methods

### Synthesis of FPC

The FPC used in this study was manufactured as previously described (McCall 2010) under good manufacturing practices conditions as a powder, dissolved in water, and packaged as a sterile solution.

### EXAFS and XANES measurements and analysis

Iron K edge (7112 eV) X-ray absorption fine structure (XAFS) measurements (encompassing both X-ray absorption near-edge structures [XANES] and extended X-ray absorption fine structure [EXAFS]) were performed at beamline location 10-BM of the advanced photon source (APS) at Argonne National Laboratory. Samples were measured in transmission mode using Si(111) monochromatic crystals using N_2_ filled ion chambers. Iron K edge XAFS measurements were conducted on powdered and aqueous solution of FPC (10 mM) and food-grade SFP, as well as the ferric standards described below. Data were analyzed using the UWXAFS package (Stern et al. 1995). Processing of the raw data, including alignment of data sets and background removal, were implemented using ATHENA (Ravel and Newville 2005). Multiple k-weight (k1, k2, and k3) fitting of each spectrum was performed using the Fourier-transformed χ(R) spectra. The χ(k) data range used for EXAFS Fourier transforms was 2.3–9.5 Å^−1^. Hanning window function with a dk value of 1.0 Å^−1^ was used to avoid truncation ripples in the Fourier transform (Newville et al. 1995). The fitting range for all the data sets was 1.0–3.5 Å.

EXAFS data analysis was based on refining theoretical EXAFS spectra of crystallographic information from well-characterized standard compounds against the experimental data. ARTEMIS (Ravel and Newville 2005) was used to fit the EXAFS data from powdered iron standards. R factor as a measure of goodness of fit and statistically significantly lower reduced Chi square values were used as criteria for improvement in the fit to justify the addition of an atomic shell to the model (Mishra et al. 2009).

### XAFS standards

Crystalline powder standards (ferric pyrophosphate, ferric acetate, ferric carbonate, and ferric citrate) were measured and used to calibrate the theoretical calculations against experimental data. Fitting of the powder standards to their known crystallographic structure reproduced the spectral features in the entire fitting range (1.0–4.2 Å). Only the paths necessary to model the solid standards were used for fitting the solution standards and the unknown iron samples.

In addition to crystalline powder standards, solution-phase standards [iron (III), ferric citrate complex, ferric pyrophosphate, and ferric desferroxamine B] were also measured.

Iron (III) was prepared by adding 100 mM iron (III) ICP standard in nitric acid at pH 2.0. To prepare aqueous ferric citrate complex, 10 mM iron (III) was added to a 50 mM citrate buffer and reacted for 1 week. Solution standards for ferric pyrophosphate and ferric desferroxamine B were prepared by dissolving the appropriate quantity of the corresponding salts in deionized water to obtain 10 mM solution for each standard. The best-fit values of the solution standards were used as the initial guess parameters of the corresponding variables for fitting the unknown iron samples.

### Infrared spectroscopy

The infrared spectrum was obtained using a PerkinElmer Spectrum One FTIR Spectrometer. The sample was prepared as a 3–3.5% dispersion in anhydrous potassium bromide.

### Ion-exchange chromatography

The anionic content of FPC was determined using a Dionex™ ICS 5000 + metal-free high-performance liquid chromatography (HPLC) system equipped with an anionic electrolytically regenerated suppressor and conductivity detector. Target anions were separated on a Dionex™ 4 × 250 mm IonPac™ AS15 IC column and AG15 Guard column using a sodium hydroxide gradient.

### Size exclusion chromatography

FPC sample was dissolved in deionized water and analyzed using an isocratic separation on a Waters UltraHydrogel Linear 300 × 7.8 mm HPLC column with refractive index detection.

## Results

### Infrared spectroscopy

**Fig. 1:**
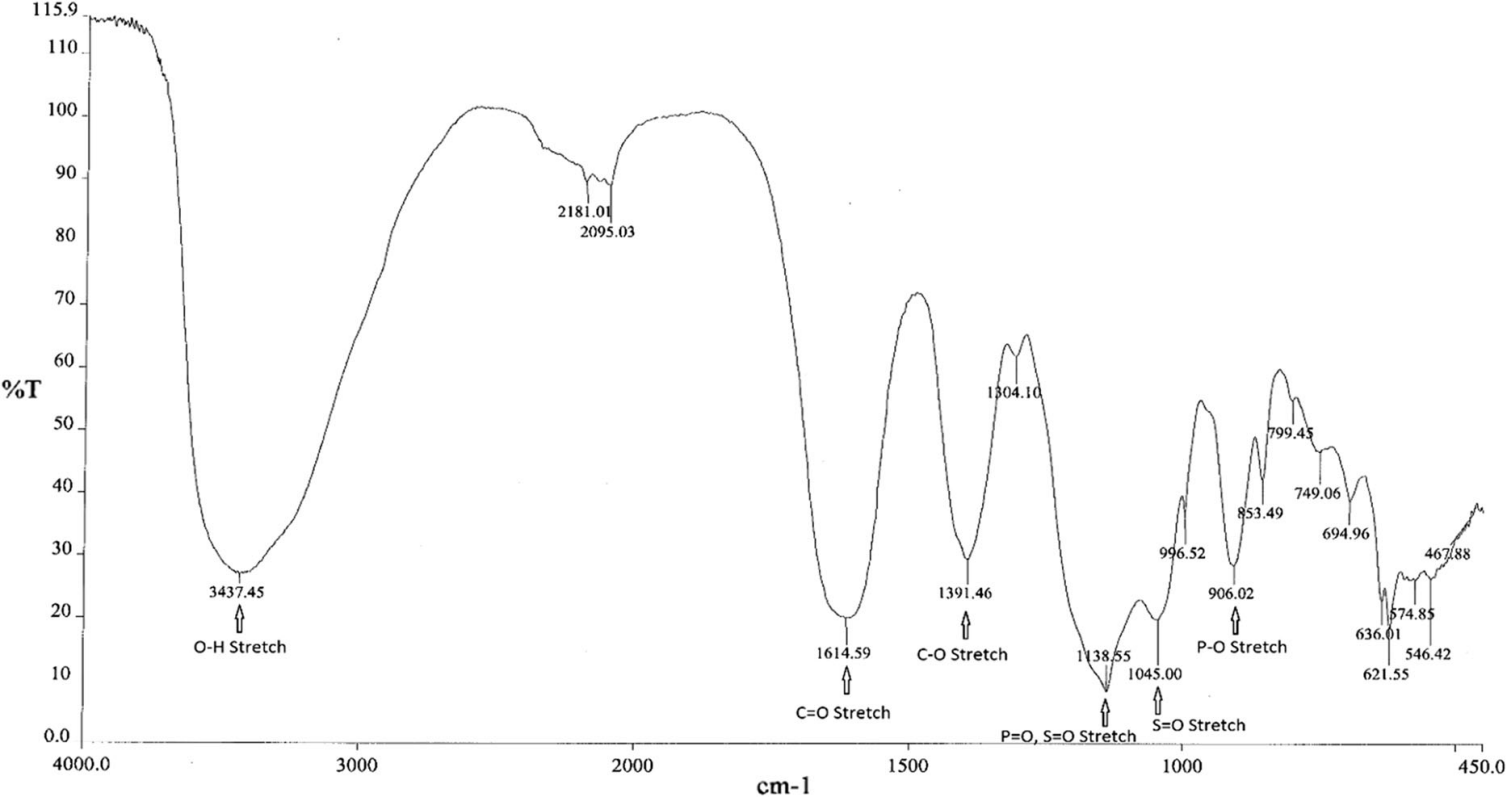
IR spectrum of FPC. IR spectrum of FPC shows characteristic peaks for the main functional groups, including the O–H stretch, C=O stretch, C–O stretch, P=O and S=O stretches, and P–O stretch

Infrared (IR) spectroscopy was used to determine the main functional groups present in FPC. Figure 1 shows a representative IR spectrum of FPC. Peak assignments and positions for FPC as well as for sodium citrate, sodium pyrophosphate, and ferric sulfate, which were used to confirm the peak assignments, are shown in Table 1.

**Table 1:** IR Peaks and assignments for FPC and standards

IR peaks (cm^−1^)				Assignment
FPC	Sodium citrate	Sodium pyrophosphate	Ferric sulfate	
3437	3259	3242	3144	O–H stretch
1615	1592			C=O stretch
1391	1420			C–O stretch
1139		1115	1126	P=O, S=O stretch
1045			1028	S=O stretch
906		926		P–O stretch

### X-ray absorption spectroscopy

XAS is a powerful structural tool that provides information on the oxidation state and short-range coordination environment of the atom(s) under study (Stern 1974). Both XANES and EXAFS were used in this study. EXAFS provides information on the short-range coordination environment of the atom under study, such as interatomic distances (R), nearest and next-nearest neighbor coordination numbers (CN) and associated mean square displacement (sigma square), while XANES provides information on the net oxidation state of the atom under probe.

### Iron standards

**Fig. 2:**
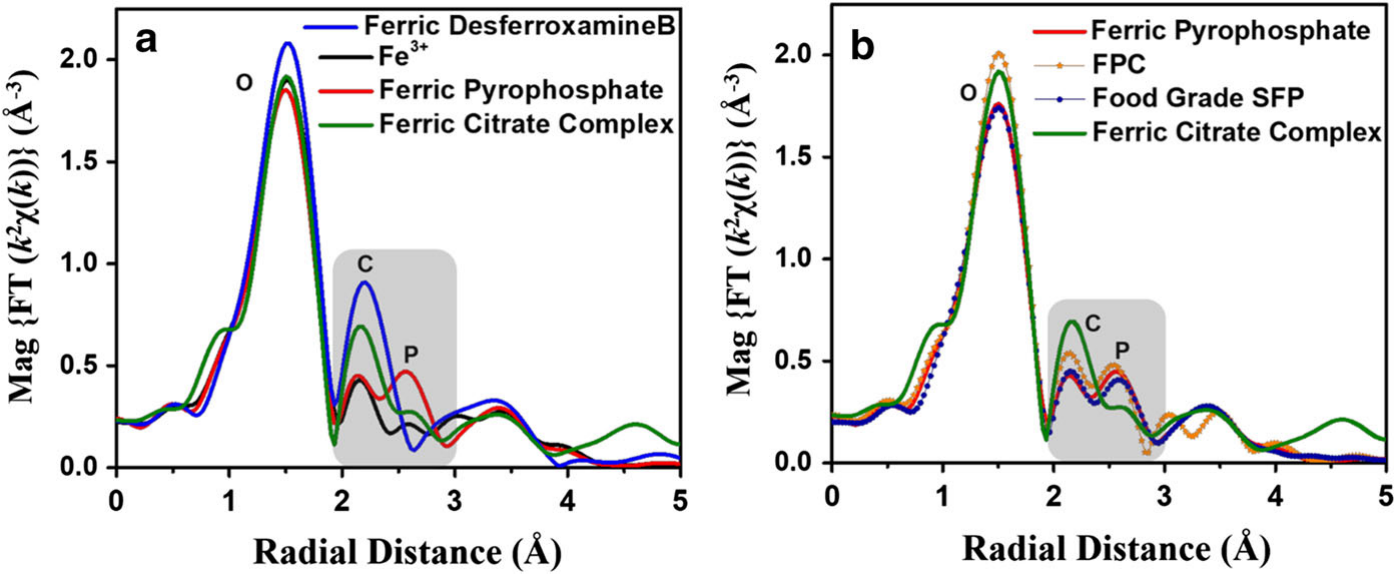
Fourier-transformed EXAFS spectra of iron standards, FPC, and SFP. Fourier-transformed EXAFS spectra for **a** iron standards (Fe^3+^, ferric pyrophosphate, ferric citrate complex, and ferric desferroxamine B) and **b** FPC, food-grade SFP, ferric citrate complex, and ferric pyrophosphate

**Fig. 3:**
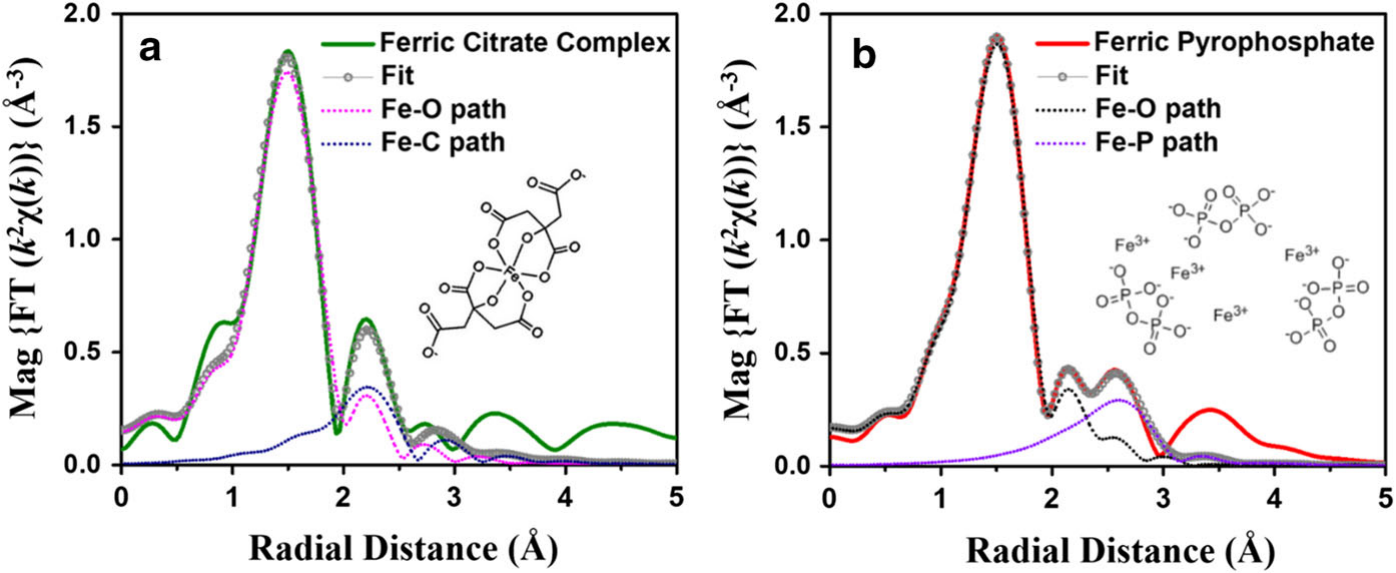
Fourier-transformed EXAFS spectra for ferric citrate and ferric pyrophosphate. Data, fit, and contributions of different signals for the magnitude of Fourier-transformed EXAFS spectra for **a** ferric citrate complex and **b** ferric pyrophosphate

Iron coordination number, interatomic distances, and mean square displacement for the iron solution standards iron (III), ferric pyrophosphate, ferric citrate complex, and ferric desferroxamine B are shown in Table 2. An overlay of the Fourier-transformed EXAFS spectra for the solution standards is shown in Fig. 2a. In these spectra, the first peak corresponds to the nearest neighbor to the iron atom, the second peak to the next nearest neighbor, and so on. The height of the peak represents the number of atoms while the peak position corresponds to the bond distance. The width and shape of the peak represent structural disorder or contribution from multiple scattering (MS). Fourier-transformed EXAFS spectra and fit for ferric citrate complex and ferric pyrophosphate, the standards used to draw conclusions on FPC and food-grade SFP, are shown in Fig. 3.

**Table 2:** Best fit values (coordination number, interatomic distances, and mean square displacement) for EXAFS analyses of iron standards, FPC, and food-grade SFP

Path	N	R (Å)	σ^2^ * 10^−3^ (Å^2^)
Fe^3+^			
Fe–O	6.0 ± 0.3	2.00 ± 0.01	7.2 ± 1.8
Ferric pyrophosphate			
Fe–O	5.8 ± 0.3	2.00 ± 0.01	5.2 ± 0.4
Fe–P	3.0 ± 0.5	3.24 ± 0.01	10.8 ± 2.2
Ferric citrate complex			
Fe–O	5.9 ± 0.3	2.00 ± 0.01	5.5 ± 0.8
Fe–C	4.2 ± 0.6	2.88 ± 0.01	5.5^a^
Ferric desferroxamine B			
Fe–O	6.0 ± 0.3	2.00 ± 0.01	5.0 ± 1.2
Fe–C/N	6.0^b^	2.82 ± 0.01	5.0^a^
Food-grade SFP^c^			
Fe–O	5.5 ± 0.3	2.00 ± 0.01	5.2 ± 0.4
Fe–P	2.8 ± 0.6	3.24 ± 0.01	10.8 ± 2.2
FPC^d^			
Fe–O	5.8 ± 0.3	2.00 ± 0.01	4.1 ± 0.3
Fe–P	2.5 ± 0.3	3.24 ± 0.01	10.8 ± 2.2
Fe–C	3.6 ± 0.6	2.90 ± 0.01	8.2 ± 2.5

^a^Set to be same as sigma square for Fe–O

^b^Set to be same as coordination number for Fe–O

^c^Food-grade SFP was modeled simultaneously with ferric pyrophosphate

^d^FPC was modeled simultaneously with ferric pyrophosphate and ferric citrate

EXAFS confirms octahedral coordination chemistry of iron (6 O atoms) in both ferric citrate complex (Fig. 3a) and ferric pyrophosphate (Fig. 3b). EXAFS analyses of the Fe–O–C and Fe–O–P iron coordination environments of ferric pyrophosphate [5.8 ± 0.3 O atoms at 2.00 ± 0.01 Å and 3.0 ± 0.5 P atoms at 3.24 ± 0.01 Å (Table 2)] are in good agreement with previously published results (Elbouaanani et al. 2002). Our results suggest that each iron center in the polynuclear pyrophosphate molecule interacts with three phosphorus atoms on average. High Debye–Waller type factor associated with the Fe–O–P bond suggests that the Fe–O–P bond acts as a bridge between repeat units of ferric pyrophosphate.

Aqueous ferric desferroxamine B, in which the iron atom is bound to three carbon and three nitrogen atoms via six oxygen atoms, was used for unambiguous assignment of the Fe–O–C signal. Since EXAFS cannot distinguish between carbon and nitrogen atom backscattering in the second shell, ferric desferroxamine B was modeled with six oxygen atoms in the first shell and six carbon/nitrogen atoms in the second shell. EXAFS modeling of aqueous ferric citrate was conducted simultaneously with ferric desferroxamine B. Results suggest that ferric citrate complex was bound with 4.2 ± 0.6 carbon atoms, suggesting a predominant Fe(citrate)_2_ binding mechanism. However, the bond distance of Fe–O–C in ferric citrate was longer (2.88 ± 0.01 Å) than that of ferric desferroxamine B (2.82 ± 0.01 Å) (Table 2). Although both standards have a monodendate binding mechanism (one O bound to one C), small variations in the Fe–O–C bond length could arise from differences in ligand environment. The shorter Fe–O–C bond length in desferroxamine B is consistent with a highly symmetric and ordered structure in which all six carbon/nitrogen atoms are part a strong inner sphere complex.

### Solid FPC and food-grade SFP

**Fig. 4:**
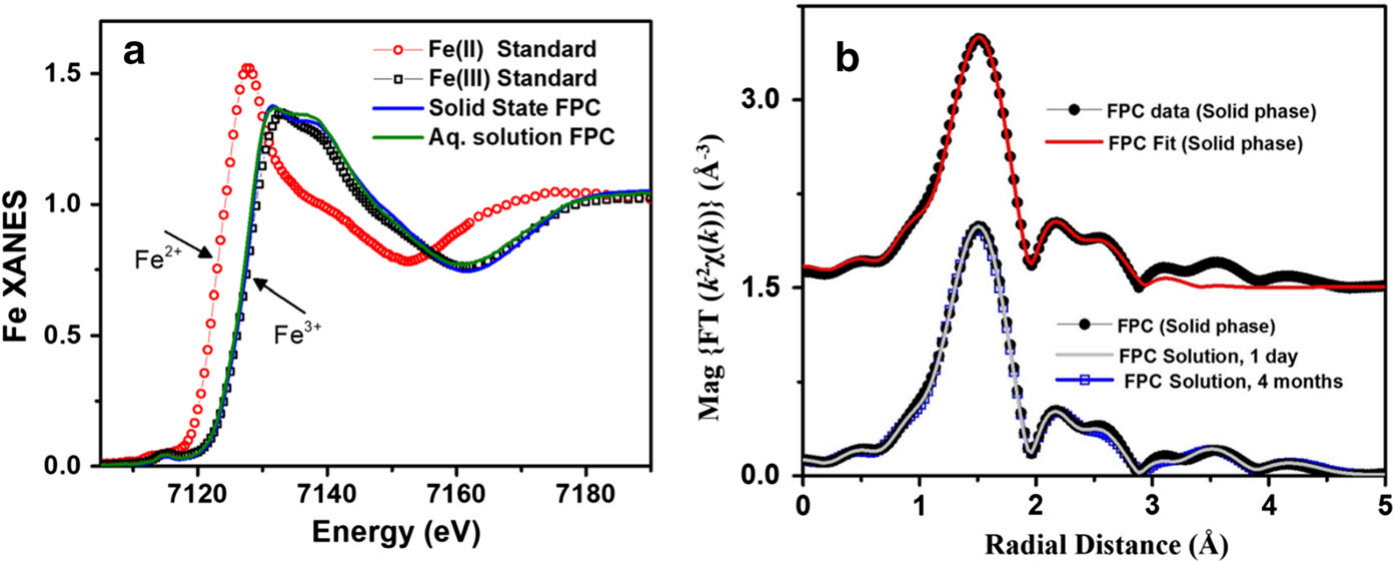
X-ray spectra of solid and aqueous iron standards and FPC. **a** XANES spectra of iron (II) and iron (III) standards as well as FPC in the solid and solution phases show that FPC consists exclusively of iron (III) and that the solid-phase structure is maintained in solution. **b** EXFAS modeling of FPC in the solid phase (top) and in solution (bottom) at Day 1 and Month 4

Iron EXAFS fitting parameters for FPC and food-grade SFP are shown in Table 2. XANES data of FPC compared to iron (II) and an iron (III) standards reveal that all the iron in FPC is present as iron (III) (Fig. 4).

Supplementary S1 Fig shows an overly of the EXAFS spectra for FPC, food-grade SFP, and ferric pyrophosphate and ferric citrate standards. While the food-grade SFP spectrum overlays nicely with the ferric pyrophosphate spectrum, the FPC spectrum demonstrates features of both the ferric pyrophosphate and ferric citrate complex spectra. A comparison of the Fourier-transformed EXAFS spectra of FPC and food-grade SFP is shown in Fig. 2b. Qualitative analysis suggests that FPC has both Fe–O–P and Fe–O–C signals, while food-grade SFP seems to have only a Fe–O–P signal. Analysis of the FPC EXAFS data reveals that iron (III) is complexed with oxygen as the nearest neighbor, with a bond length of 2.00 ± 0.01 Å and with phosphorus and carbon atoms as next nearest neighbors, with bond lengths of 3.24 ± 0.01 Å and 2.90 ± 0.01 Å, respectively (Fig. 2b; Table 2).

In contrast to FPC, food-grade SFP appears bound to 2.8 ± 0.6 P atoms (Table 2). Iron bound citrate ligation was not detected in food-grade SFP, consistent with the observation that the EXAFS data of food-grade SFP are nearly identical to the ferric pyrophosphate data.

Although qualitative inspection suggests that the carbon signal in FPC is smaller than that in ferric citrate complex (Fig. 2b), visual inspection of EXAFS data could be deceptive due to the interference of overlapping signals in a mixed coordination environment. Quantitative EXAFS analysis shows that the phosphorus signal in FPC interferes destructively with the carbon signals, causing it to appear smaller than the Fe(citrate)_2_ signal. Quantitative EXAFS modeling of FPC shows that the iron center is bound to 2.5 ± 0.3 phosphorus and 3.6 ± 0.6 carbon atoms (Table 2), suggesting an average molecular complex with one pyrophosphate and two citrate ligands.

**Fig. 5:**
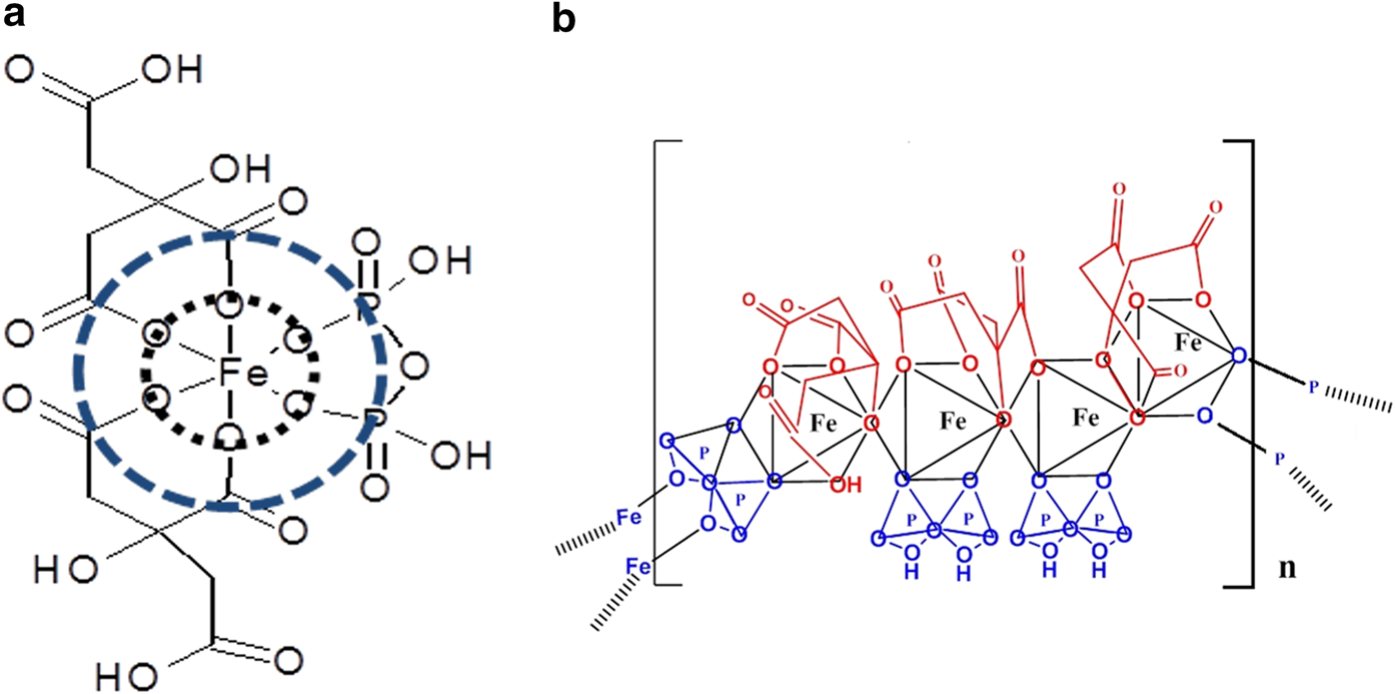
Proposed primary structure of FPC. **a** Proposed coordination of structure of FPC. Dotted lines represent first and second coordination shells. **b** Repeat unit of FPC

Based on these data, a coordination structure of FPC can be proposed in which iron (III) is complexed with one pyrophosphate and two citrate molecules **(**Fig. 5). Four of the oxygen atom bonds to iron (III) come from the citrate anion while two come from pyrophosphate. The coordination number of 6 for iron (III) is completely satisfied by pyrophosphate and citrate oxygen atom donors, with pyrophosphate acting as a bridging ligand between the iron center. There are no vacant or labile aquated coordination sites on the iron (III).

### Aqueous FPC

EXAFS spectrum of solution-state FPC overlay nearly identically with the solid-phase spectrum (Fig. 4b), suggesting that the iron coordination environment does not change between solid and solution phases. XAFS (Fig. 4b) and ion chromatography show that the solid-state structure is stable for up to four months in solution at room temperature.

### FPC stoichiometry and repeat unit

FPC stoichiometry was determined by ion-exchange HPLC. S2 Fig shows a representative chromatogram. The chromatogram contains four major peaks, one at 6.1 min, corresponding to the sulfate anion, one at 7.8 min, corresponding to the citrate anion, and one at 8.8 min, corresponding to the pyrophosphate anion. A smaller peak at 7.1 min corresponds to the phosphate anion. Table 3 shows the composition of FPC as determined by ion chromatography. Ion chromatography confirms the Fe:citrate:pyrophosphate ratio of 4:3:3. Elemental analysis and IR spectra demonstrate that FPC coprecipitates as a solid with the composition Na_12_[(Fe^3+^)_4_(cit^4−^)_3_(pyrophosphate^4−^)_3_]Na_12_(SO_4_)_6_ 7H_2_O (data not shown). The structure for this repeat unit, which can be defined as four iron atoms bridged by citrate anions, is shown in Fig. 5b. The molecular weight (MW) of the repeat unit is 1312 Da. X-ray powder diffraction (XRPD) data indicate that FPC is an amorphous or nanocrystalline material (data not shown), making it difficult to obtain a precise MW. However, membrane diffusion data suggest a MW of approximately 1300 Da, consistent with FPC dissociating to a single repeat unit as shown in Fig. 5b.

**Table 3:** Chemical composition of ferric pyrophosphate citrate

Ion	Percentage
Iron	8
Citrate	19
Pyrophosphate	18
Phosphate	< 1
Sulfate	25–28

## Discussion

Ferric pyrophosphate citrate is a new member of the class of iron salts knows as SFP. Compared to other soluble ferric pyrophosphate complexes, FPC has improved solubility (> 1000 mg/mL) and stability and is the first pharmaceutical iron product suitable for parenteral administration that is noncolloidal and does not contain a carbohydrate moiety. In this study, we present the solid- and solution-phase structures of FPC. IR and HPLC analyses of FPC confirm the presence of the expected anions, citrate and pyrophosphate, as well as phosphate and sulfate. It should be noted that these methods will show all anions present while XAS analyses will only show iron-bound anions. EXAFS data show that only pyrophosphate and citrate ions are complexed to the iron atom. Based on these data, we conclude that FPC is a purely iron (III) compound, with the iron atom complexed with one pyrophosphate and two citrate molecules. Complexation of iron (III) with two citrate molecules increases solubility and stability. Previous studies of ferric citrate have demonstrated that citrate can act as a (4-) anionic tetradentate ligand to bridge two iron (III) ions (Bino et al. 1998; Shweky et al. 1994). XAFS data show that the solid-state structure of FPC remains intact in the solution phase and is stable in solution for up to 4 months. The differences in chemical structure between SFP and colloidal IV iron–carbohydrate formulations account for differences in metabolism and pharmacokinetics of these compounds upon parenteral administration (Geisser and Burckhardt 2011; Gupta et al. 1999, 2015; Pratt et al. 2017).

FPC is a unique iron compound and a promising new means to provide iron to hemodialysis patients. The clinical pharmacology of FPC shows dose proportional pharmacokinetics up to the maximal iron binding capacity of serum. Additionally, FPC does not increase hepcidin at the doses used to maintain hemoglobin in chronic hemodialysis patients (Pratt et al. 2017). Two multicenter, randomized, placebo-controlled, phase 3 clinical trials demonstrated that FPC safely replaces iron losses and maintains hemoglobin levels without increasing iron stores in patients undergoing chronic hemodialysis (Fishbane et al. 2015). In these patients, the high solubility of FPC allows delivery through the dialysate as it can efficiently cross the dialyzer membrane. In clinical trials, delivery of FPC via the dialysate significantly reduced erythropoiesis-stimulating agent dose and IV iron needed to maintain hemoglobin concentration without promoting oxidative stress. (Gupta et al. 2015). In 2015, FPC was approved by the US FDA for delivery via hemodialysis solutions to replace ongoing iron losses, thereby maintaining hemoglobin concentrations in hemodialysis-dependent patients with CKD (Rockwell Medical 2018). Anaphylaxis has not been observed in over 600,000 doses of FPC administered during clinical trials and post-marketing (Data on file, Rockwell Medical Inc, Wixom MI, USA); while other iron compounds have been reported to cause anaphylaxis at a rate of 20 or more cases per million doses administered (Wang et al. 2015). Whether the unique chemical structure of FPC, including lack of a carbohydrate moiety, are responsible for the growing evidence of enhanced safety remains to be determined.

## Electronic supplementary material

Below is the link to the electronic supplementary material.

Supplementary material 1 (DOCX 463 kb)
