# Supplementary material for: Physicochemical characterization of ferric pyrophosphate citrate
Source: Biometals. 2018 Oct 15;31(6):1091–9. doi: 10.1007/s10534-018-0151-1 (PMC6245090; doi:10.1007/s10534-018-0151-1)
Supplement: Supplementary file 1 — Supplementary material 1 (DOCX 463 kb) [file 10534_2018_151_MOESM1_ESM.docx]

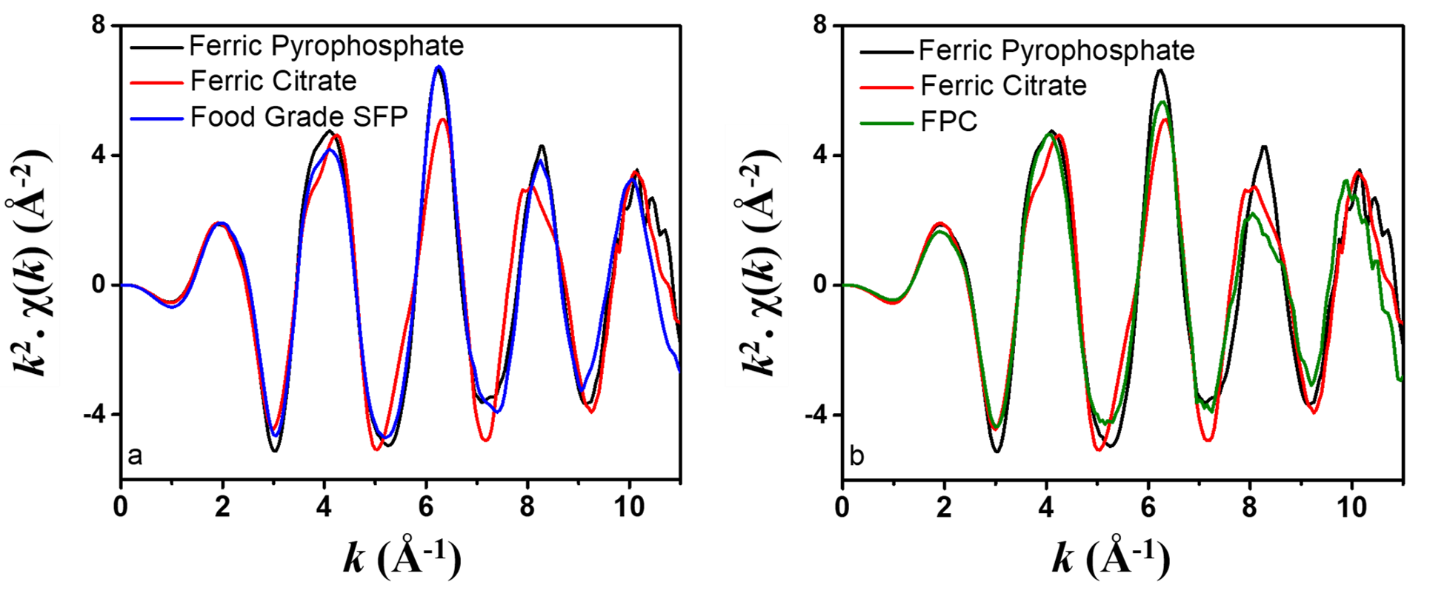


**S1 Fig. EXAFS data for iron standards, FPC, and food-grade SFP.** EXAFS data comparing ferric pyrophosphate and ferric citrate with a) food grade SFP and b) FPC.


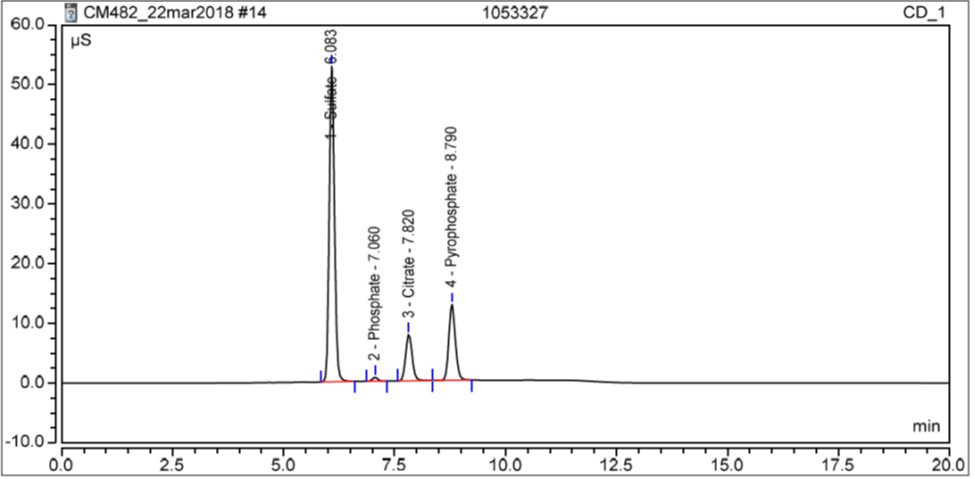


**S2 Fig. HPLC chromatogram for FPC.** Chromatogram of FPC by Ion-Exchange HPLC.
